# JACLNet:Application of adaptive code length network in JavaScript malicious code detection

**DOI:** 10.1371/journal.pone.0277891

**Published:** 2022-12-14

**Authors:** Zhining Zhang, Liang Wan, Kun Chu, Shusheng Li, Haodong Wei, Lu Tang

**Affiliations:** State Key Laboratory of Public Big Data, College of Computer Science and Technology, Guizhou University, Guiyang, China; Hanyang University, KOREA, REPUBLIC OF

## Abstract

Currently, JavaScript malicious code detection methods are becoming more and more effective. Still, the existing methods based on deep learning are poor at detecting too long or too short JavaScript code. Based on this, this paper proposes an adaptive code length deep learning network JACLNet, composed of convolutional block RDCNet, BiLSTM and Transfrom, to capture the association features of the variable distance between codes. Firstly, an abstract syntax tree recombination algorithm is designed to provide rich syntax information for feature extraction. Secondly, a deep residual convolution block network (RDCNet) is designed to capture short-distance association features between codes. Finally, this paper proposes a JACLNet network for JavaScript malicious code detection. To verify that the model presented in this paper can effectively detect variable JavaScript code, we divide the datasets used in this paper into long text dataset DB_Long; short text dataset DB_Short, original dataset DB_Or and enhanced dataset DB_Re. In DB_Long, our method’s *F*1 − *score* is 98.87%, higher than that of JSContana by 2.52%. In DB_Short, our method’s *F*1-*score* is 97.32%, higher than that of JSContana by 7.79%. To verify that the abstract syntax tree recombination algorithm proposed in this paper can provide rich syntax information for subsequent models, we conduct comparative experiments on DB_Or and DB_Re. In DPCNN+BiLSTM, *F*1-*score* with abstract syntax tree recombination increased by 1.72%, and in JSContana, *F*1-*score* with abstract syntax tree recombination increased by 1.50%. *F*1-*score* with abstract syntax tree recombination in JACNet improved by 1.00% otherwise unused.

## Introduction

With the development of front-end technology, more and more applications began to provide services in the form of web applications, leading to a sharp increase in the number of web-based applications. As a cross-platform language, JavaScript can be embedded decentrally and executed dynamically [1]. Its convenience has made it the language of choice for front-end development, but it also poses risks to web applications [2]. An attacker can inject malicious JavaScript code into a web page to perform malicious acts, such as spreading Trojan viruses, accessing sensitive user information and mining data [3].

In a real-world environment, the length of the JavaScript code to be detected can be very short (just a few kilobytes) or very long (more than 1MB), and there is a wide range between them. The existing deep learning detection methods cannot accurately detect too short or too long JavaScript codes in feature learning. This is because in model training, the initial weight of features of excessively short JavaScript codes is relatively small, which is easy to be weakened in the process of model training. The main features of excessively long JavaScript codes usually run through the whole JavaScript code. This requires the ability of the model to capture long-distance association features to capture overlong JavaScript code features.

The shallow convolutional neural network has a simple structure that can effectively capture the association features over short-distances, but not the information loss of the association features over long-distances [4]. Currently, deep convolutional neural networks are connected by residual networks to prevent the gradient from exploding or disappearing when the convolutional depth is deepened [5]. However, they are separated by several convolutional blocks when the residual block is connected. In JavaScript code, the file size is smaller than that of the text. When these deep convolutional neural networks are used, the association feature is weakened at short-distances. The cyclic neural network trains each word using the sequential method, which results in a longer training and recognition time than other models. Moreover, the weighting information is shared in the cyclic neural network, which means that the model cannot take into account the important part of the contextual information [6, 7].

Aiming at the problem that the existing methods can’t capture the correlation feature of variable distance between codes, this paper proposes an adaptive code length network JACLNet to detect malicious JavaScript codes. In this paper, small convolution kernels are used to obtain short-distance correlation features between codes (associations between 9 and 21 word bits can be felt), because compared with large convolution kernels, small convolution kernels can feel more subtle relations between word bits. Residual network is used for connection in the convolution operation to prevent feature weakening after the convolution operation. In order to obtain the long-distance association and semantic information, after RDCNet operation, this paper uses the BiLSTM network to obtain the association information between the short-distance association features obtained by RDCNet. Finally, Transfrom is used to assign different weights to the long-distance management features obtained by BiLSTM. The main contributions of this paper are as follows:

1. To provide rich syntax information for subsequent feature extraction, this work first preprocesses JavaScript code, converts the processed code into an abstract syntax tree using the tool Esprima, and then recombines and renames the subtrees in the abstract syntax tree to achieve code improvement;
2. In this paper, we design a convolution block RDCNet, which consists of residual network and convolutional neural network, which can capture the correlation feature of the short-distance between codes.
3. In this paper proposes an adaptive code length neural network JACLNet. The network can extract malicious code signatures from JavaScript files that are too long or too short.

The remainder of this article is organised as follows. Section 2 presents the methods currently used to detect malicious JavaScript code. Section 3 describes the method presented in this paper. Section 4 describes the dataset, experimental setup and results of this experiment. Section 5 summarises the work in this paper and gives an outlook on future research directions.

## Related work

This section will briefly discuss three related topics: external convolutional neural networks, deep convolutional neural networks and deep learning in malicious JavaScript code detection.

### Shallow convolutional neural network

The shallow convolutional neural networks use several convolutional neural networks to perform specific tasks [8]. In short-distance association feature detection, Kim et al. [9] used one layer of convolutional neural network to obtain local features in the initial phase. It uses static word embedding and non-static word embedding as input to the convolutional layer to extract features. Finally, it tests several short text field datasets and verifies that a single-layer CNN still performs well on certain tasks. Zhang et al. [10] used different word embedding and model parameters based on Kim-CNN [9] and finally selected the optimal word embedding and parameters in TextCNN. Liu et al. [11] modified Kim-CNN [9] twice. First, they use dynamic max-pooling to capture more fine-grained features from different document regions. Second, a hidden bottleneck layer is inserted between the pooling layer and the output layer to learn a compact text representation, which reduces the model size and improves the model performance. Rie et al. [12] did not use the pre-trained low-dimensional word vector as input to the CNN model, but applied CNN directly to high-dimensional text data to learn the information embedded in small texts.

### Deep convolutional neural network

The Deep Convolutional Neural Network uses multiple convolutional layers to accumulate deep information [13]. To solve the problem that shallow convolutional neural networks cannot capture association features over long-distances. Zhang et al. [14] proposed a character-level convolutional neural network that uses character-level text as the source signal and employs a one-dimensional convolutional neural network to process it. The results show that the Deep Character Level Convolutional Neural Network is effective on large datasets. Conneau et al. [6] propose a very deep character-level convolutional neural network (VDCNN) that uses alternating connections of convolution operations for acquisition. VDCNN performs well on extensive training data but relatively poorly on small training data. Zhang et al. [5] used 9-layer and 29-layer deep convolutional neural networks at the character level and 1-layer and 2-layer neural networks at the word level to conduct comparative experiments on large datasets. The results show that the very shallow layer of word-level CNN is more accurate and faster than the very deep layer of character-level CNN when dealing with long-distance associations, and that the expressiveness of word-level CNN is stronger than that of character-level CNN. In order to find an efficient deep word-level CNN model, Johnson et al. [15] re-examined the word-level CNN based on Zhang’s [5] experimental results and found a deep pyramid structure of convolutional neural network (DPCNN) with low complexity that can capture features of long-distance association. The above deep convolutional neural network methods perform better on large datasets and worse on small datasets than shallow convolutional neural networks. Kaliyar et al. [16] proposed a new deep convolutional neural network, FNDNet, which added two convolutional layers and two fully connected layers based on the TextCNN model. Experimental results show that this model is better than other models in recognising short texts.

### Deep learning of JavaScript

In recent years, deep learning methods have begun to be applied in security [17, 18], and are widely used in JavaScript malicious code. Recurrent neural network (RNN), long short memory (LSTM) and gate recurrent unit (GRU), have been widely applied to JavaScript malicious code due to its strong performance in processing text structures. Yong et al. [19] used LSTM to obtain context information and solve the problem of long term sequence dependency. However, if the text sequence is too long, LSTM has the feature weakening problem. Song et al. [20] used BLSTM for feature extraction to solve the Yong-LSTM [19] problem. Since the weighting information is shared in the recurrent neural network, the model cannot consider the important part of contextual information. Yong et al. [21] added Attention model after the BiLSTM to obtain important information in the BiLSTM to solve the problem that the recurrent neural network could not obtain important contextual information. Since the recurrent neural network consumes resources during training and testing, a convolutional neural network or the method that combines the recurrent neural network with the convolutional neural network is used to solve this problem. Rozi et al. [22] used the combination form of DPCNN and BiLSTM for feature extraction. Since the residual connection of DPCNN is only at the beginning and the end, such connection would weaken the short-distance correlation feature in JavaScript. Huang et al. [23] used TextCNN to obtain local features between codes based on word vectors trained by dynamic word embedding. Yong et al. [24] added a GRU model based on graph convolution GCN to obtain information from graph data while reducing parameters, and used the GRU model to remember the hidden information of neighbouring nodes and the hidden information in the iterative process.

The literature review above shows that the external neural convolutional network has a simple structure and can only capture association features over short-distances, not association features over long-distances. The current convolutional layer of the deep neural network is relatively deep, and several convolutional blocks are separated when residual blocks are connected. In JavaScript code, the average file is only a few hundred 10KB in size. When these deep convolutional neural networks are used, the association features are weakened over short-distances. Existing methods for detecting malicious JavaScript code have hardly explored the association features at different distances. For this reason, this paper proposes an JACLNet neural network for JavaScript malicious code detection. This article approach is described in the next section.

## Proposed method

**Fig 1:**
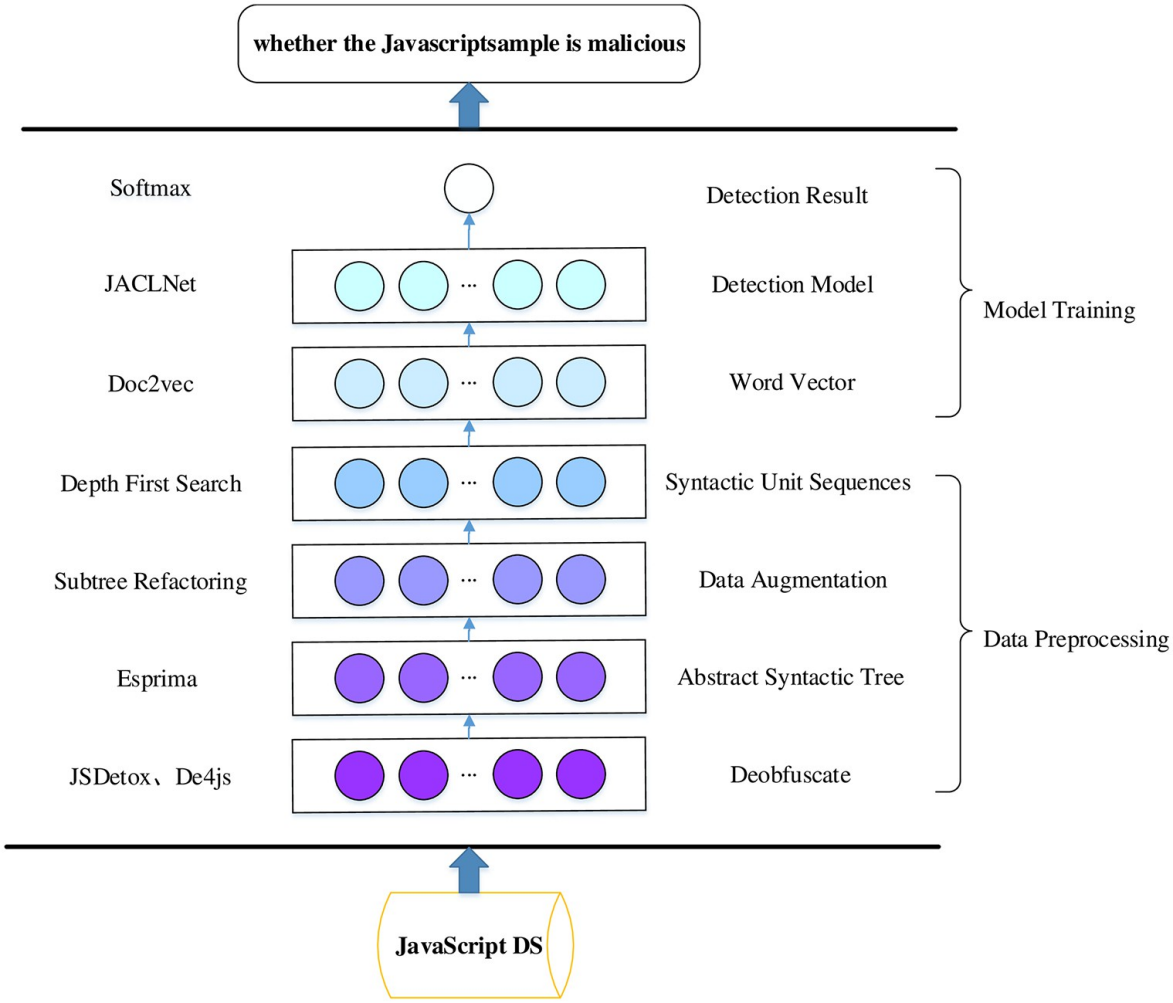
This paper proposes a JavaScript malicious code detection method framework.

Fig 1 shows the overall architecture of this paper. In this paper, JavaScript malicious code detection is divided into two parts. The first part is data preprocessing. This paper uses the de-obfuscation tool to de-obfuscate the obfuscated code. Use tree-shaking [25] in Rollup to remove the dead code.The processed JavaScript code is transformed into an abstract syntax tree, and statement blocks and methods are obtained through breadth search. Data enhancement is achieved by replacing the order of statement blocks and methods and changing variable names. The syntax unit sequence is obtained by deep search traversal of the modified abstract syntax tree. The second part is the construction of the detection model. In this paper, Doc2vec [26] is used to generate the word vector and serve as the embedding layer of the model word vector. Use JACLNet as the JavaScript malicious code detection model. The methods used in this article are described in detail below.

### Data preprocessing

In data preprocessing, a distinction can be made between code deobfuscation, code to abstract syntax tree, recombination of the abstract syntax tree and abstract syntax tree to grammar sequence.

#### Deobfuscate

In model training, if the obfuscated JavaScript codes are directly extracted from features, the obtained model will prefer the detection of obfuscated JavaScript, and the false negative phenomenon may occur for the malicious codes in non-obfuscated JavaScript. In order to solve this problem, this paper uses the deobfuscation tool to deobfuscate the confused JavaScript first and then extract features. In order to select a better deobfuscation tool, this paper compared the deobfuscation tools JSDetox, De4js, JSnice and Bejson [27–30], and the comparison results are shown in Table 1.Through comparison, JSDetox and JSnice are selected as the deobfuscation tools in this paper.

**Table 1:** Deobfuscation tools and obfuscation category tables.

Deobfuscation category		Deobfuscation tool			
		JSDetox	De4js	JSnice	bejson
String manipulation obfuscation		✓	✓	✓	✓
Numeric substitution obfuscation		✓	✓	✓	✓
Encoding obfuscation	Standard code obfuscation	✓	✓	✓	✓
	Custom encoding obfuscation				
Code logic obfuscation	The dead code				
	Structure of obfuscation	✓			

For dead code, this article uses Rollupjs tree-shaking to remove dead code. Since Rollup statically analyses JavaScript code to exclude unused code, this method saves time compared to the V8 engine and removes dead code as effectively as V8.

#### Cord augmentation and syntax sequence generation

The abstract syntax tree (AST) is an abstract representation of the syntactic structure of source code. It represents the syntactic structure of a programming language as a tree, where each node in the tree represents a structure in the source code [31]. Currently, AST is used to accurately represent this kind of opaque JavaScript code. Since AST can represent necessary functional structures of the code, such as scopes, expressions or declarations, it avoids redundancy by omitting unnecessary syntax details, such as whitespace, pun action markers or comments, even after the parsing process does not affect the original functionality of the code.

**Algorithm 1** Abstract syntax tree reorganization

**Input:** *Ast*Abstract syntax tree class object

**Output:** *newAst*Abstract syntax tree reassembles class objects

1: **function** recomAST(*Ast*)

2:  *mapVar* ← {}

3:  *cordC*, *cordNC* ← []

4:  *indexC*, *indexNC* ← 1

   //getBlock() gets functions and statement blocks.

5:  *block* ← getBlock(*Ast*)

6:  **for** *i* = 1 to *len*(*block*) **do**

7:   *value* ← *block*[*i*][′*name*′]

8:   **if** *value* **in** *mapVar* **then**

9:    *block*[*i*][′*name*′] ← *mapVar*[*value*]

10:   **else**

   //RanVar() randomly generates string variables.

11:    *newVar* ← RanVar()

   //ifEx() Check whether newVar exists.

12:    **while** ifEx(*mapVar*, *newVar*) **do**

13:     *newVar* ← RanVar()

14:    **end while**

15:    *mapVar*[*value*] ← *newVar*

16:    *block*[*i*][′*name*′] ← *newVar*

   //ifBlock() determines whether a block or function is included.

17:    **if** ifBlock(*block*[*i*][′*type*′]) **then**

18:     *newBlock* ← reAST(*block*[*i*][′*type*′])

19:     *cordC*[*indexC*] ← *newBlock*

20:     *indexC* ← *indexC* + 1

21:    **end if**

22:    *cordC*[*indexC*] ← *newBlock*

23:    *indexC* ← *indexC* + 1

  //ifDef() determines if it is a statement definition.

24:    **if** ifDef(*block*[*i*]) **then**

25:     *cordC*[*indexChage*] ← *block*[*i*]

26:     *indexC* ← *indexC* + 1

27:    **else**

28:     *cordNC*[*cordNC*] ← *block*[*i*]

29:     *indexNC* ← *indexNC* + 1

30:    **end if**

31:   **end if**

32:  **end for**

33:  //ranList() randomly arranges the list.

34:  **return** ranList(*cordC*) + *cordNC*

35: **end function**

Since Esprima [32] is a powerful, standards-compliant ECMAScript parser, it has 69 different node types, including expression nodes, type nodes, class nodes, declaration nodes, etc. Various code fragments are mapped to corresponding nodes, which contain extensive syntax information. In this article, the Esprima tool is used to transfer JavaScript code into the AST structure.

**Fig 2:**
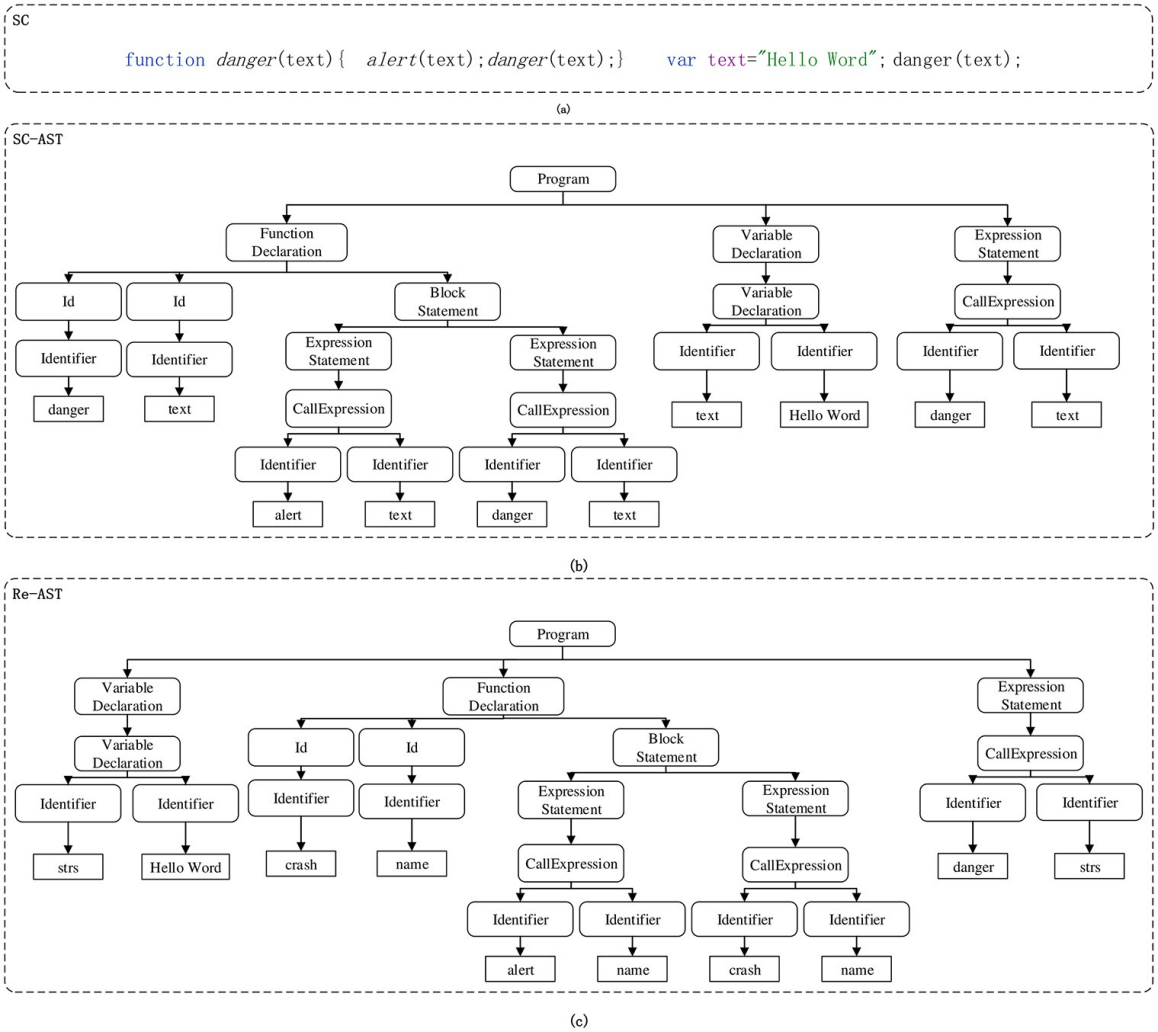
Abstract syntax tree reorganization.

To improve the generalisability of the model, this article improves the code, mainly by restructuring the abstract syntax tree(Just change the position of the function definition subtree) and renaming variables. Fig 2(a) is the source code, Fig 2(b) is the abstract syntax tree of Fig 2(a) and 2(c) is the abstract syntax tree reconstructed from Fig 2(a). We place the function definition subtree between the traversal of the “text” definition and the call to the function “danger”, and replace the variable name “text” with “strs”, the function name “danger” with “crash”, and the function parameter name “text” with “name”. The method of changing variable name and subtree recombination can realize code enhancement and provide rich feature information for subsequent feature extraction. Algorithm 1 gives the implementation process of code enhancement. We first obtain each statement, statement block and method node of the AST abstract syntax tree through breadth traversal search, and then iterate the results of a breadth traversal search, rename variables, and reorganize definition statements, statement blocks and methods.

**Fig 3:**
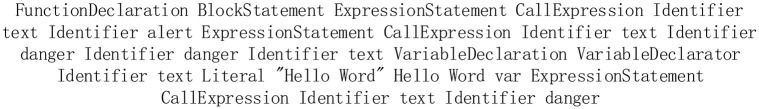
Syntactic sequence units.

To better extract the features of the JavaScript cord, we use a deep-first traversal algorithm to determine the sequence of syntax units by traversing the nodes of each statement in the abstract syntax tree. Fig 3 is the sequence of grammatical units obtained by depth-first traversal algorithm for Fig 2(b). After obtaining the sequence of grammar units, this paper uses a Doc2vec model for word vector training, and the trained word vector is used as the input of the detection model embedding layer.

### Detection model construction

**Fig 4:**
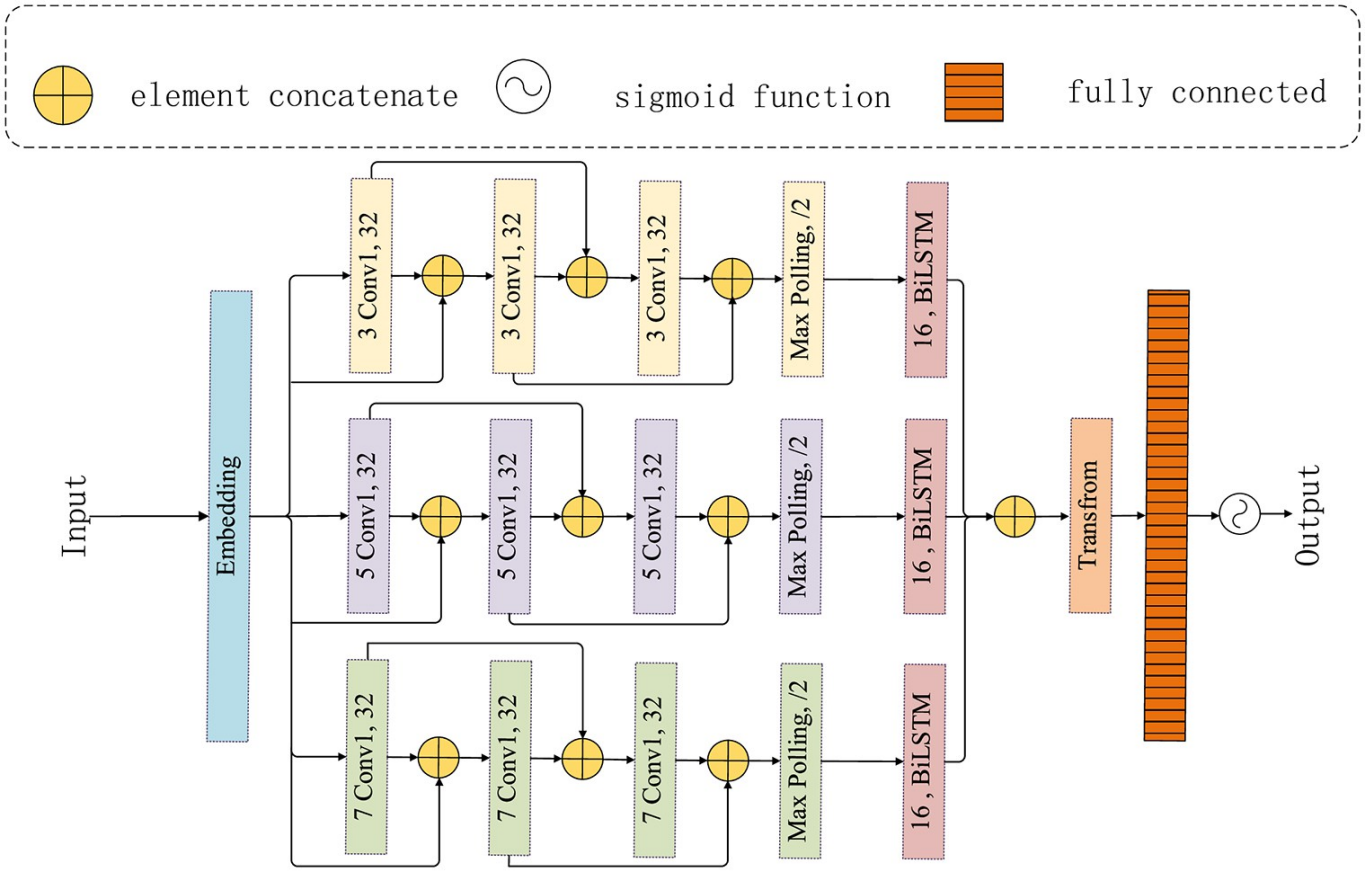
JACLNet framework diagram.

JACLNet is illustrated in Fig 4. The first layer is the word embedding layer, followed by the RDCNet network of three different convolutional kernels and the parallel operation of BiLSTM, and finally the data is aggregated into a vector through concatenate. Operate with Transfrom to get the output of the JACLNet model. This paper RDCNet network using different convolution kernels for capturing features, mainly because of the different convolution kernels can feel the link between the different word features, this paper used convolution kernels is a convolution kernel (3,5,7), respectively, this is because a convolution kernel relative to the big convolution kernels can feel more subtle relationship between lexeme. Since RDCNet can feel information of 21 bits at most, it cannot obtain long-distance correlation features. In order to obtain long-distance features, BiLSTM network was added after RDCNet, and BiLSTM network was used to obtain the association information between short-distance association features obtained by RDCNet, and then the long-distance association features and semantic information between codes were obtained. Since the weight information of BiLSTM is shared, the importance degree of the long-distance correlation features obtained by BILSTM is basically the same. In order to highlight the important information in the long-distance association features, this paper added Transfrom after BiLSTM operation to add different weight information to the long-distance association features obtained by BiLSTM, so as to highlight the important features.

#### RDCNet

**Fig 5:**
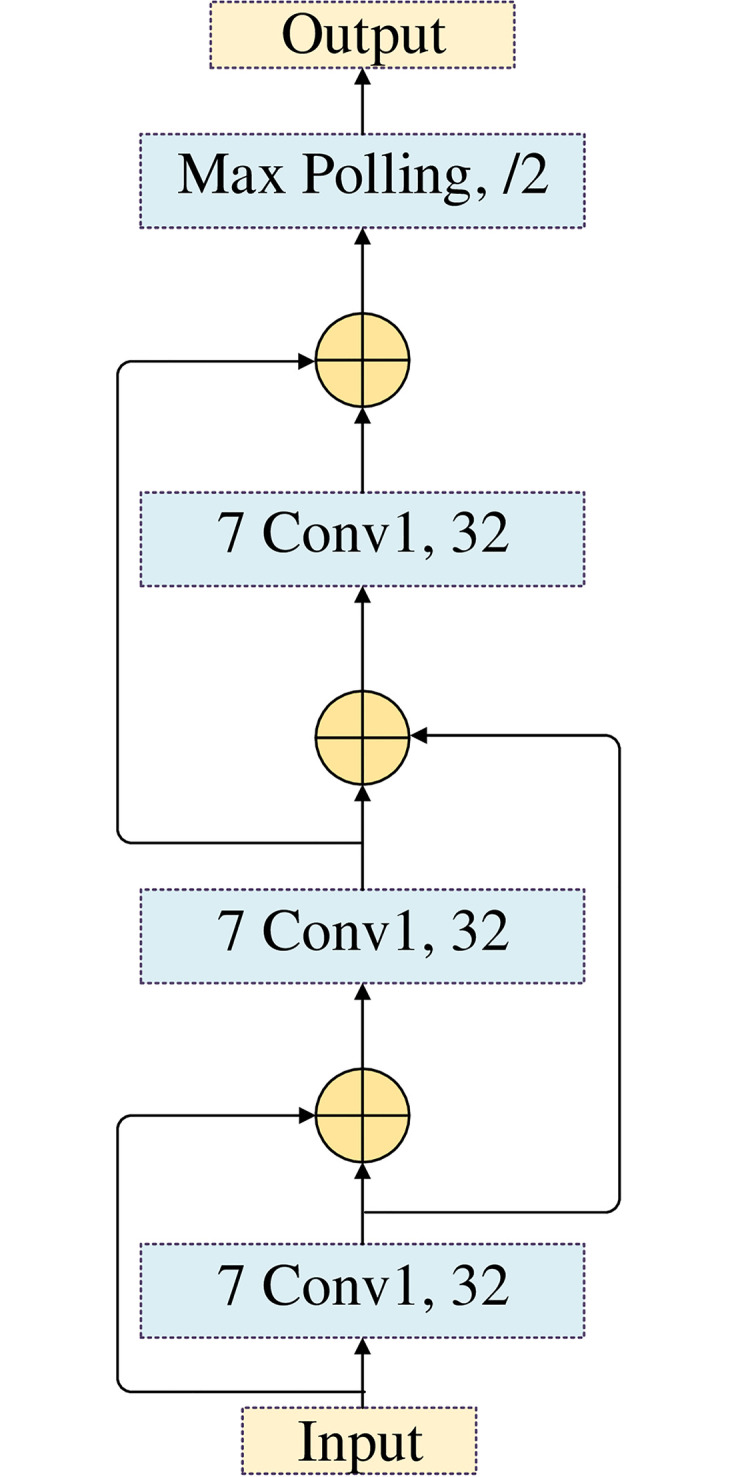
RDCNet framework diagram.

RDCNet is shown in Fig 5. RDCNet consists of three identical convolution operations and pooling operations. In order to obtain the correlation feature between words, the convolution operation in this paper uses equal length convolution. Since the number of inputs and outputs of the isometric convolution is the same, after the convolution operation of the convolution kernel with size n, the context information of each word bit of the input sequence and its left and right $\frac{\left(n-1\right)}{2}$ will be compressed into this word bit and output. When the convolution operation is deepened, each word output will contain more contextual information, so as to achieve the effect of long-distance correlation feature capture. In this article, the number of filters is fixed at 32 because increasing the number of filters greatly increases the computation time, not the accuracy [21]. The same convolution kernel is used for operation (as JavaScript codes are generally hundreds of KB, the small convolution kernel used in this paper is 3, 5 and 7), and the information of 21 word bits can be sensed after three times of convolution. In the pooling operation, a max pooling with a size of 3 and a stride of 2 is used. After max pooling, the length of the sequence is compressed to half of the original size.

In the convolution operation, the weight of each layer usually starts with a very small value, which causes the output value to approach 0 as the depth is deepened, and then the short-distance association information is lost. At the same time, since the affine matrix (the connecting edge between the two layers) is approximately multiplied in the deep network, the network is prone to gradient explosion or dispersion problems in the training process. To solve these problems, this paper uses the residual connection method, which takes the output of the upper layer network and the output of the current layer network simultaneously as input to the next layer network, to solve the problem of short-distance association feature information loss, gradient explosion and disappearance.

Let *x*_*i*_ ∈ *R*^*k*^ represent the *k*-dimensional word vector corresponding to the *i* word in the sentence.

$$\begin{array}{c} x_{1:n}=x_{1}⊕x_{2}⊕\cdots ⊕x_{n} \end{array}$$ (1)

Where ⊕ is the join operator. Let *x*_*i*:*i*+*j*_ represent the word *x*_*i*_, *x*_*i*+1_, …, *x*_*i*+*j*_. In RDCNet, the convolution operation uses filter *w* ∈ *R*^*hk*^, which is applied to *h* word Windows to generate a new feature. For example, feature *c*_*i*_ is generated by a window consisting of the word *x*_*i*:*i*+*h*−1_ and is represented as follows:

$$\begin{array}{c} c_{i}=f\left(w\cdot x_{i:i+h-1}+b\right) \end{array}$$ (2)

Here *b* ∈ *R* is a bias term and *f* is the activation function (e.g. Relu). The filter is applied to each word window in sentence {*x*_1:*h*_, *x*_2:*h*+1_, …, *x*_*n*−*h*+1:*n*_} to produce a feature map. Its feature graph is shown as follows:

$$\begin{array}{c} c=[c_{1},c_{2},\ldots ,c_{n-h+1}] \end{array}$$ (3)

When the above convolution operation is operated once, the feature result $c_{1}^{{}^{\prime }}$ is obtained, and then the residual connection between $c_{1}^{{}^{\prime }}$ and the input *x*_*i*_ is made to obtain the feature $c_{1}^{{}^{\prime \prime }}$. The operation is as follows:

$$\begin{array}{c} c_{1}^{{}^{\prime \prime }}=x_{i}+c_{1}^{{}^{\prime }} \end{array}$$ (4)

Then carry out the above convolution and splicing operations. When the feature $c_{3}^{{}^{\prime }}$ is obtained after two convolution operations, then splice $c_{3}^{{}^{\prime }}$ with the result $c_{1}^{{}^{\prime }}$ of the first convolution to get a *c*_*t*_, and pool *c*_*t*_. MaxPooling mainly takes the maximum value $\hat{c_{t}}=\text{max}\left\{c_{t}\right\}$. Through the above operations, the output feature *y*_*ddc*_ of RDCNet is finally obtained.

#### BiLSTM

**Fig 6:**
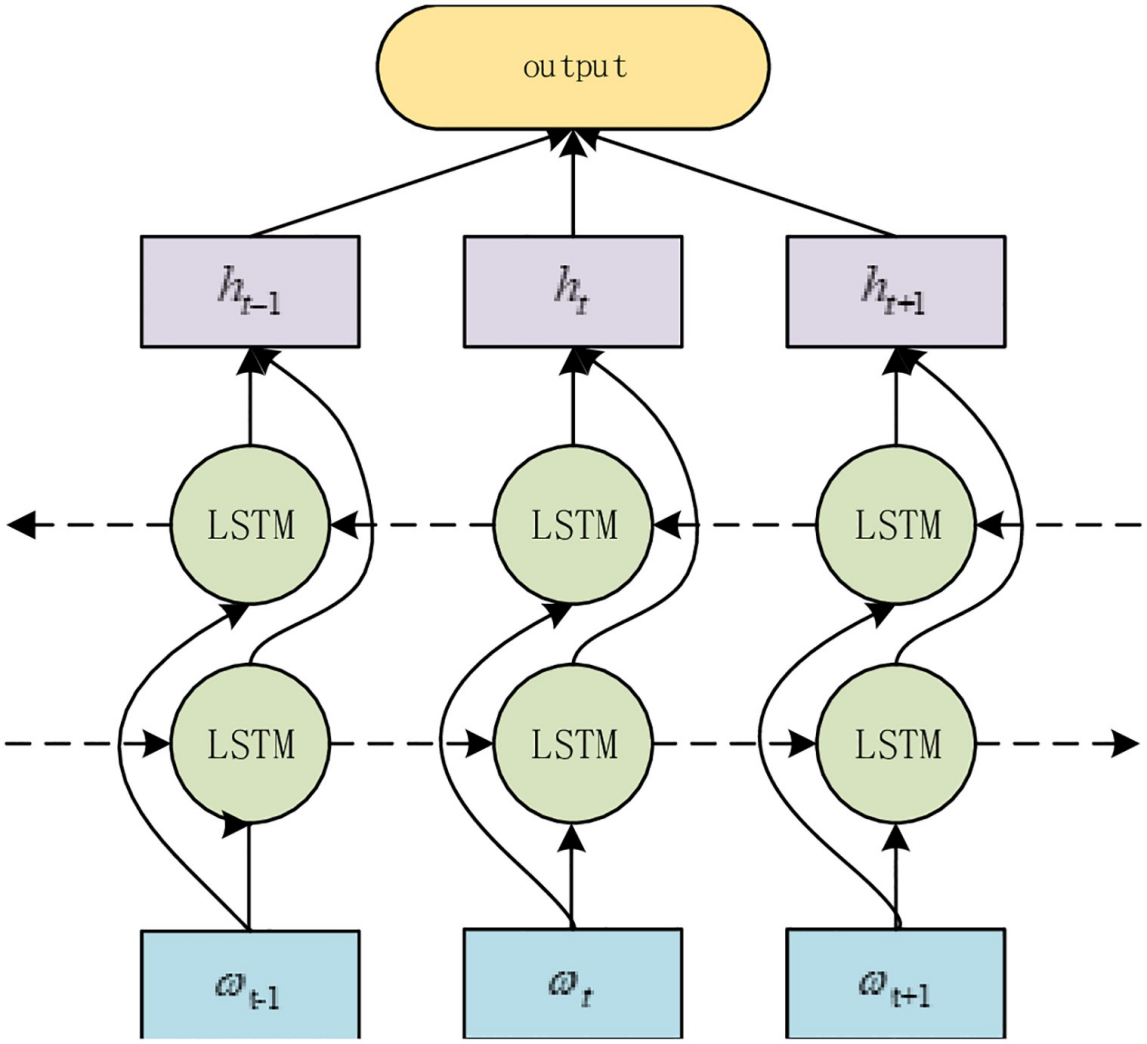
BiLSTM framework diagram.

As the above RDCNet can feel information with a maximum length of 21 bits (when the convolution kernel is 7), long-distance correlation features cannot be obtained. In order to obtain long-distance features, BiLSTM was added after RDCNet to obtain the long-distance correlation features and semantic information between features extracted by RDCNet. BiLSTM structure is shown in Fig 6.

BiLSTM is trained separately by two-way LSTM, and finally the two, information is fused to obtain context-related information. The BiLSTM training formula is as follows:

$$\begin{array}{c} \vec{h_{f}}=\vec{LSTM}\left(L_{n}\right),n\in [1,1300] \end{array}$$ (5)

$$\begin{array}{c} \vec{h_{b}}=\vec{LSTM}\left(L_{n}\right),n\in [1,1300] \end{array}$$ (6)

$$\begin{array}{c} h=[\vec{h_{f}},\vec{h_{b}}] \end{array}$$ (7)

Where, $\vec{h_{f}}$ represents the forward LSTM result, *L*_*n*_ is the NTH word, $\vec{LSTM}\left(L_{n}\right)$ represents LSTM forward training method. $\vec{h_{b}}$ represents the backward LSTM result, $\vec{LSTM}\left(L_{n}\right)$ represents the LSTM backward training method. In this paper, the number of word vectors is set to 1300, so the range of N is *n* ∈ [1, 1300]. *h*represents the final training result of BiLSTM.

In this paper, the result *h*_1_,*h*_2_ and *h*_3_ were obtained after three times of BiLSTM operation, and then the output of RDCNet+BiLSTM network was obtained by stitching the following formula:

$$\begin{array}{c} y_{bilstm}=[h_{1};h_{2};h_{3}] \end{array}$$ (8)

#### Transfrom

BiLSTM can obtain long-distance association features, but BiLSTM uses the shared weight globally, which leads to the same importance degree in the acquired long-distance association features. In order to highlight important information in long-distance association features, this paper adds Transfrom after BiLSTM operation to add weight information to long-distance association features obtained by BiLSTM and highlight important features.

**Fig 7:**
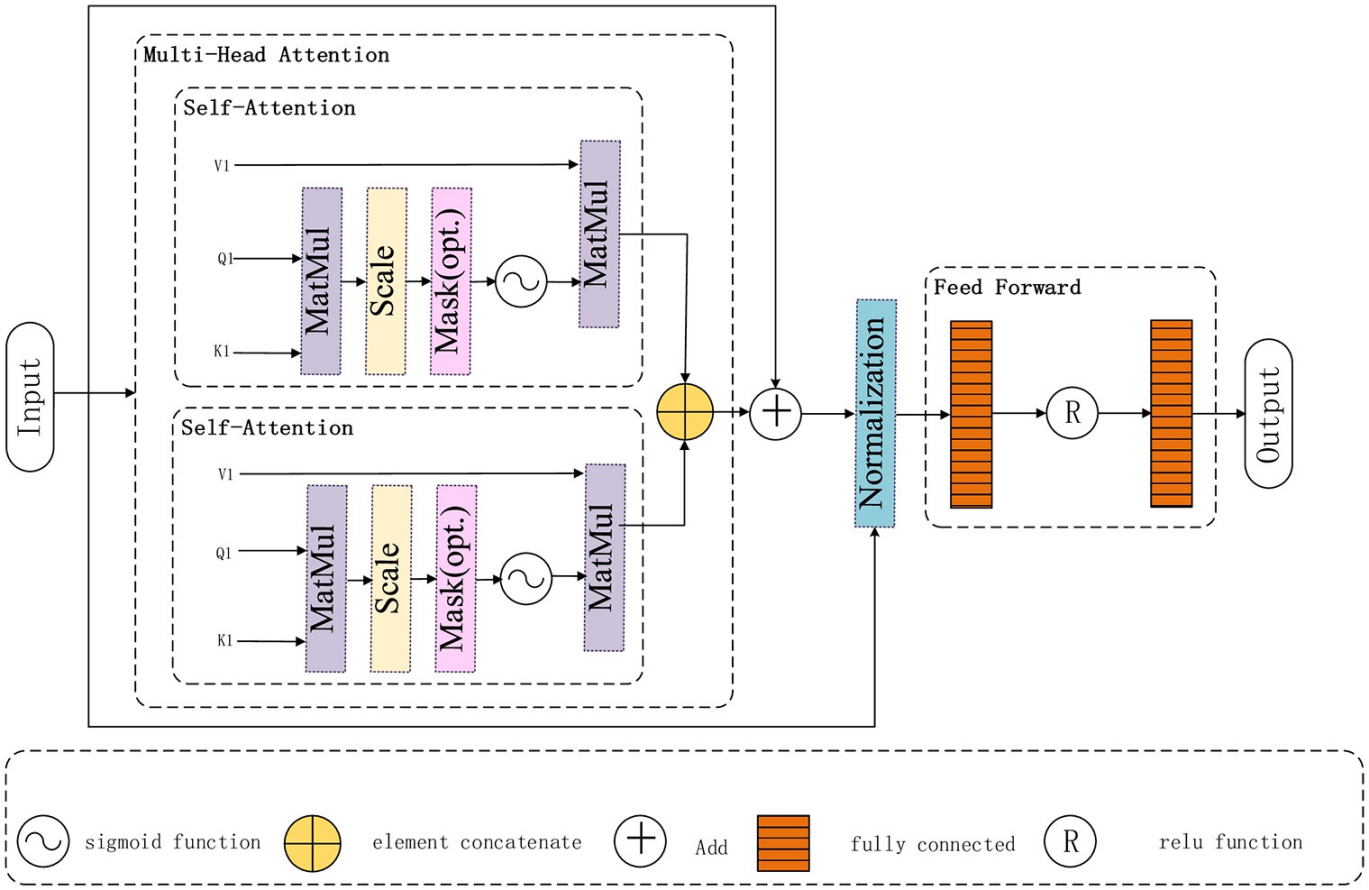
Transfrom framework diagram.

The Transfrom structure of this article is shown in Fig 7. The Multi-Head Attention network is composed of two Self-Attention networks, which mainly relearn *y*_*bilstm*_ and assign different weights to each word. Its operation is as follows:

$$\begin{array}{c} Q=y_{bilstm}\cdot w_{q} \end{array}$$ (9)

$$\begin{array}{c} K=y_{bilstm}\cdot w_{k} \end{array}$$ (10)

$$\begin{array}{c} V=y_{bilstm}\cdot w_{v} \end{array}$$ (11)

Where *w*_*q*_, *w*_*k*_ and *w*_*v*_ represent the weights corresponding to Q, K and V, respectively. After getting Q, K, V, the value of Self-Attention *Attention*(*Q*, *K*, *V*)is calculated by the following formula, which is the weight value of each word.

$$\begin{array}{c} Attention\left(Q,K,V\right)=softmax\left(\frac{Q\cdot K^{T}}{\sqrt{d_{k}}}\right)\cdot V \end{array}$$ (12)

Where *d*_*k*_ is the number of columns of Q,K matrix, and the inner product of Q and K divided by $\sqrt{d_{k}}$ is to prevent the inner product of Q and K from becoming too large. After the above Attention operation twice, the results *Att*_1_ and *Att*_2_ are obtained. Through concatenate, the output *y*_*add*_ of Multi-Head Attention is obtained. Its operation is as follows:

$$\begin{array}{c} y_{add}=[Att_{1}:Att_{2}] \end{array}$$ (13)

After the weight of each word is obtained, the residual connection Add is used for connection to prevent network degradation. The operation is as follows:

$$\begin{array}{c} y_{add}=Attention\left(Q,K,V\right)+y_{bilstm} \end{array}$$ (14)

Through Layer Normalization, the activation value of each layer is normalized to facilitate subsequent weight enhancement. Its operation is as follows:

$$\begin{array}{c} y_{norm}=LayerNorm\left(y_{add}+y_{bilstm}\right) \end{array}$$ (15)

Although the weight of words has been calculated in Multi-Head Attention, the results may not be strong, so Feed Forward is used to enhance the weight of words. The result of the model in this paper is *y*_*j*_*acnet*. Its operation is as follows:

$$\begin{array}{c} y_{jacnet}=max\left(0,y_{norm}\cdot w_{1}+b_{1}\right)\cdot w_{2}+b_{2} \end{array}$$ (16)

Where, *w*_1_ and *w*_2_ represent the weight information of the first fully connected layer and the second fully connected layer in the Feed Forward, and *b*_1_ and *b*_2_ represent the offset of the first fully connected layer and the second fully connected layer in the Feed Forward.

## Experiments

### DataSet description

Malshare [33] is a public malware repository that contains data uploaded by users who have been attacked by malware. It contains data of the latest attacks. HynekPetrak’s malicious code on github [34], which was mostly collected between 2015 and 2019, represents a pattern of past malware attacks. To enable the model to recognize multiple attacks, Malshare and HynekPetrak’s malicious code on github were chosen as the source of this malicious code dataset. Benign code, this article uses Alexa to rank the top 500 benign websites(Since Alexa shut down the site in May 2022, the benign site used for this article was posted on github) [35]. Mainly because Alexa’s top 500 sites are frequently-visited sites that can serve as proxies for benign code. In the end, we collected 34,718 pieces of malicious code and 17,321 pieces of benign code from the network. In this work, MD5 is used to encrypt the code content to remove duplicate code. We use Esprima tool to detect if there are syntax errors in JavaScript code and remove the code with syntax errors in this work. Finally, 32604 malicious codes and 8532 benign codes were identified. Finally, to unify the number of malicious codes and benign codes, 8500 malicious codes and benign codes were selected in this paper respectively.

In order to verify that the proposed method can adapt the text association length, we divide the collected datasets into long text sequence dataset DB_Long(The length of the text sequence after converting to AST is greater than 5000), short text sequence dataset DB_Short(The length of the text sequence after converting to AST is less than 1000) and train dataset DB_Train.

In order to enrich the semantic feature information, this paper uses subtree recombination technology to perform three recombination operations for each datum, and finally obtains 25500 malicious and benign recombination codes respectively.

This paper constructs five datasets, the first is the original dataset DB_Or; The second is the subtree recombination of the original dataset DB_Re; The third is the long text sequence dataset DB_Long; The fourth is the short text sequence dataset DB_Short; The fifth is the training dataset DB_Train. The amount of data is shown in Table 2.

**Table 2:** JavaScript dataset.

Name	Type	#JS
DB_Or	benign	8500
	malicious	8500
DB_Re	benign	25500
	malicious	25500
DB_Long	benign	1902
	malicious	1647
DB_Short	benign	1423
	malicious	1939
DB_Train	benign	5175
	malicious	4914

### Measurement metrics

This paper uses *Accuracy*, *Recall*, *Precision*, *FPR*, *F*1-*score* and Matthews correlation coefficient(MCC) to evaluate the JACLNet network proposed in this paper. Generally, the validity of a model is evaluated by *Accuray*, which is expressed as follows:

$$\begin{array}{c} Accuracy=\frac{N_{true}}{N_{totle}} \end{array}$$ (17)

Where, *N*_*true*_ represents the number of samples correctly predicted, and *N*_*totle*_ represents the total number of samples participating in the training. Since *Accuracy* cannot reflect the effect of malicious code detection in malicious code detection, this paper uses *Recall*, *FPR* and *Precision* to supplement. *Recall* represents the proportion of the malicious samples in the predicted results in the real malicious samples, *FPR* represents the ratio of the number of false positive examples to the number of all actual negative examples and *Precision* represents the proportion of the correctly predicted malicious samples in the predicted results [36]. Its representation is as follows:

$$\begin{array}{c} Recall=\frac{TP}{TP+FN} \end{array}$$ (18)

$$\begin{array}{c} Precision=\frac{TP}{TP+FP} \end{array}$$ (19)

$$\begin{array}{c} FPR=\frac{FP}{FP+TN} \end{array}$$ (20)

Where, *TP* represents the number of samples that detect malicious code as malicious code, *FN* represents the number of samples that detect malicious code as benign code, and *FP* represents the number of samples that detect benign code as malicious code.

In order to take into account *Accuracy*, *Recall*, *FPR* and *Precision*, this paper uses *F*1-*score* for evaluation, which is expressed as follows:

$$\begin{array}{c} F1\text{-}score=\frac{2\times Precision\times Recall}{Precision+Recall} \end{array}$$ (21)

In order to prevent *F*1-*score* from being overly optimistic when data is unbalanced, Matthews correlation coefficient (MCC) is added to assist *F*1-*score* in the evaluation, and its expression is as follows:

$$\begin{array}{c} MCC=\frac{TP\times TN-FP\times FN}{\sqrt{\left(TP+FP)\times (TP+FN)\times (TN+FP)\times (TN+FN\right)}} \end{array}$$ (22)

### Model uncertainty analysis

The uncertainty analysis of the model is an important index to analyze the credibility of the predicted results of the model, so before the comparison experiment, the uncertainty analysis of the model is carried out in this paper. In order to verify the uncertainty of the model, two methods are used in this paper. The first method is to use the Monte Carlo Dropout method. By randomly zeroing the output neurons of each layer of the model, this method analyzes the ability of the model to resist overfitting and the uncertainty of the model [37]. The second approach is the Model ensemble approach. The method takes several random samples from the dataset, trains the models separately, and then synthesizes the inference results of these models to analyze the uncertainty of the model [38].

The variance is how discrete the data is. In this paper, by calculating the variance of the probability of the model prediction, the dispersion degree of the model prediction results can be obtained. By analyzing the dispersion degree of the model detection results, the uncertainty analysis results of the model can be obtained. The variance formula is as follows

$$\begin{array}{c} S^{2}=\frac{\sum_{i=1}^{n}{\left(x_{i}-\bar{x}\right)}^{2}}{n} \end{array}$$ (23)

Here, *x*_*i*_ represents the predicted probability value, $\bar{x}$ represents the average value of the predicted data, and *n* represents the total number of predicted data. In general, the result of variance indicates how “surprised” the model is to see the predicted result. The lower the variance value, the more certain the model is in its prediction, that is, the lower the uncertainty of the model. The higher the variance value, the more uncertain the model’s prediction of the data, and the higher the uncertainty of the model.

**Fig 8:**
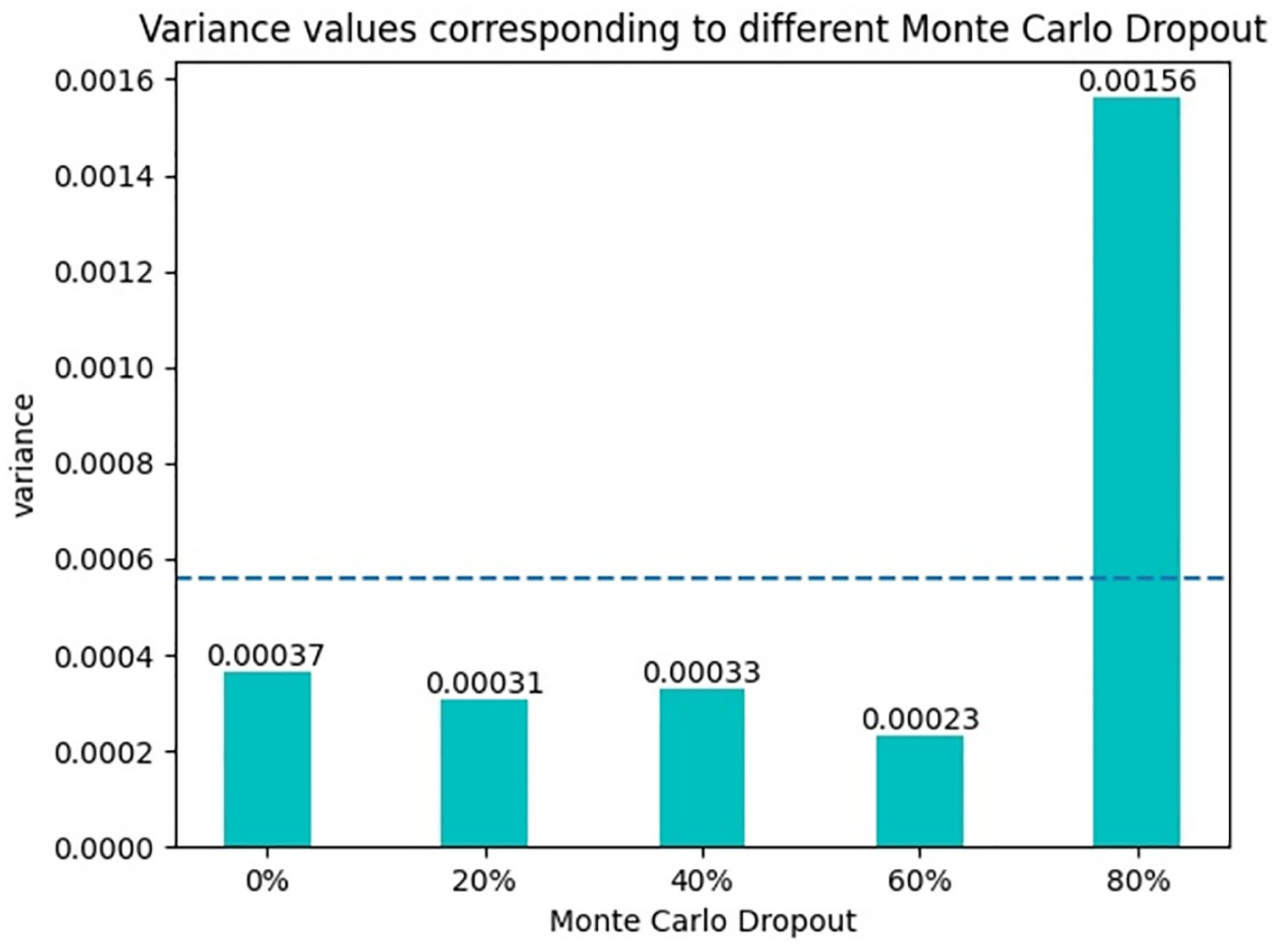
Variance values corresponding to different Monte Carlo Dropout.

In the Monte Carlo Dropout method, the Dropout technique is added to each layer of the model to randomly set the neurons to 0 according to the specified probability. In this paper, 0%, 20%, 40%, 60% and 80% are selected for experiments, and Droput is turned on during training and testing. The calculated variance results are shown in Fig 8. In Dropout Settings with different values, the average variance is 0.00055. When Dropout is set to 60%, the model has the lowest variance value of only 0.00023. When Dropout is set to 80%, the model has the highest variance value, but it is only 0.00156, which is 0.00133 different from that of 40%. By using different probabilities to zero the neurons in each layer of the model, it can be seen that the discretization degree of the model detection results is low, and the gap between them is small.

**Fig 9:**
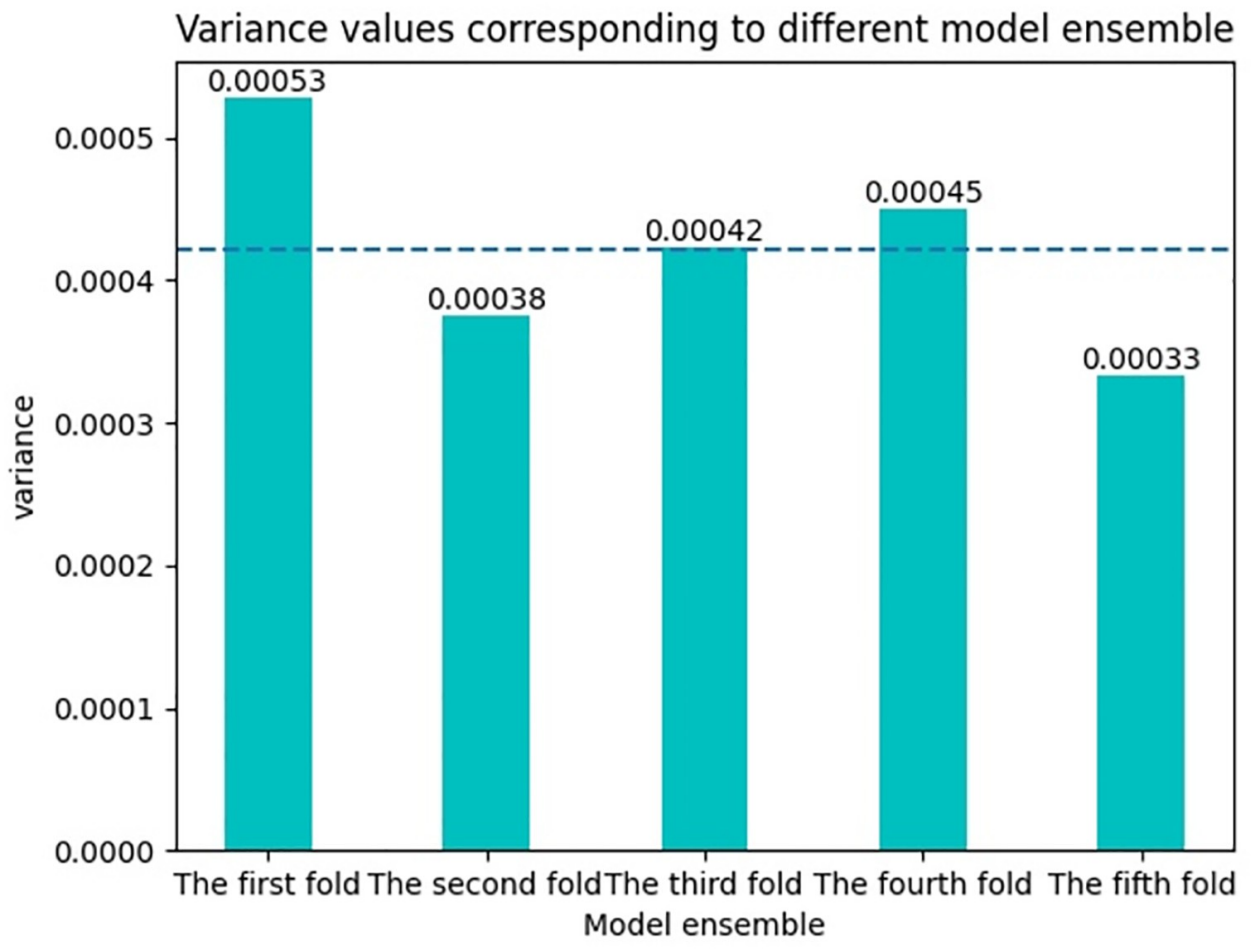
Variance values corresponding to different model ensemble.

In the model ensemble method, this paper uses the 5-fold cross validation method to ensure that each training and testing are not the same dataset. The comparison results of variance values of each model checking result are shown in Fig 9. In the 5 fold cross validation method, the average variance is 0.00042, and the difference between the minimum variance and the maximum variance is 0.00002. By comparison, it can be seen that the variance gap between the model and the probability values of detection results on different test sets is very small.

The text model JACLNet is evaluated by the two methods mentioned above. Through experiments, it is found that the probability variance values detected by the model are very low, which indicates that the model in this paper has relatively low uncertainty and high reliability of detection results.

### Experimental analysis

**Fig 10:**
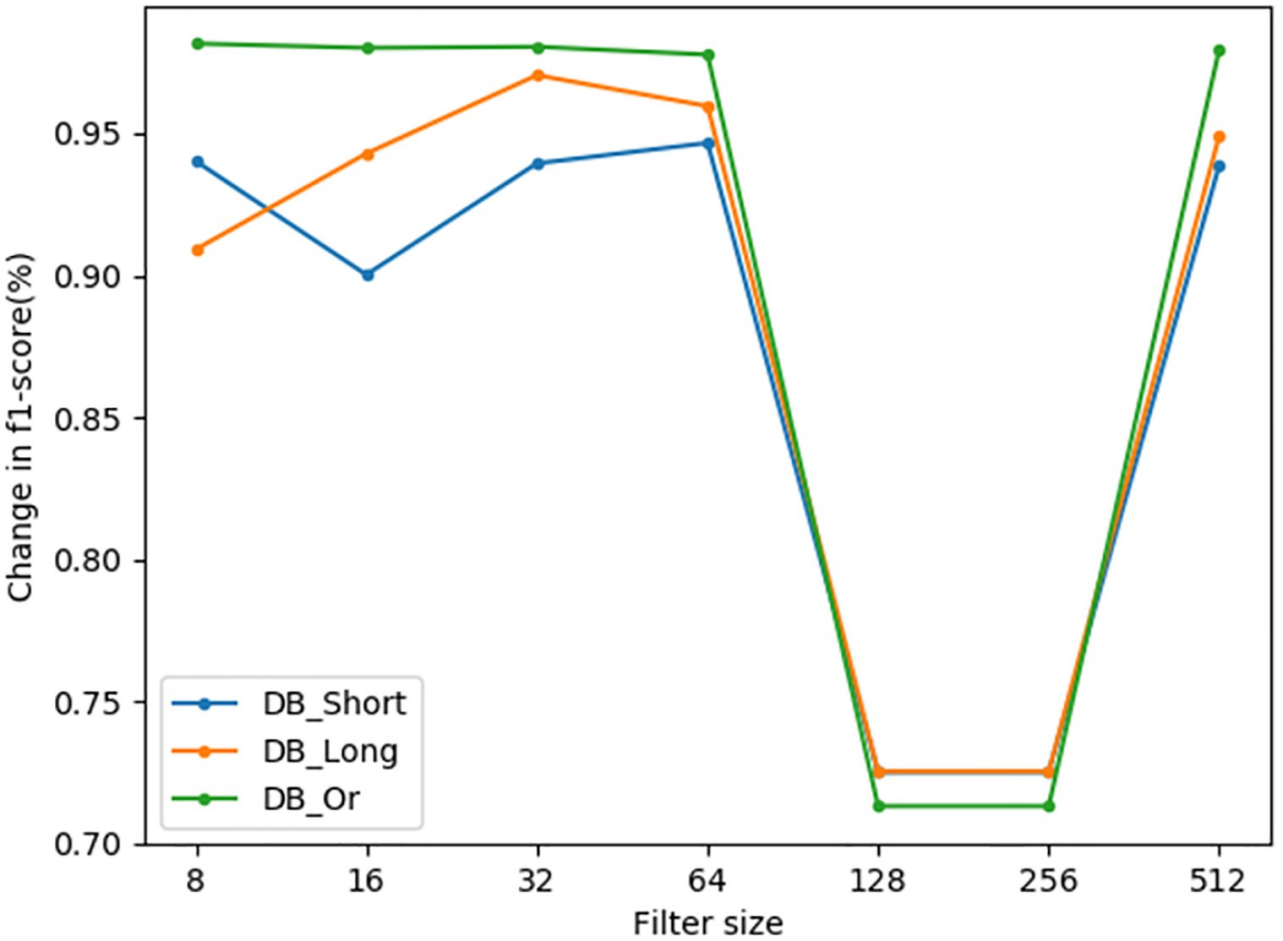
The effect of filter size.

Firstly, the effect of filter on model detection is discussed. We set the filter size to 8, 16, 32, 64, 128, 256 and 512 respectively, of experiments, and the experimental results are shown in Fig 10. Through comparison, it can be found that in DB_Or, DB_Long and DB_Shor datasets, the model detection effect is best when the filter is set to 32, so the filter in this paper is set to 32.

**Fig 11:**
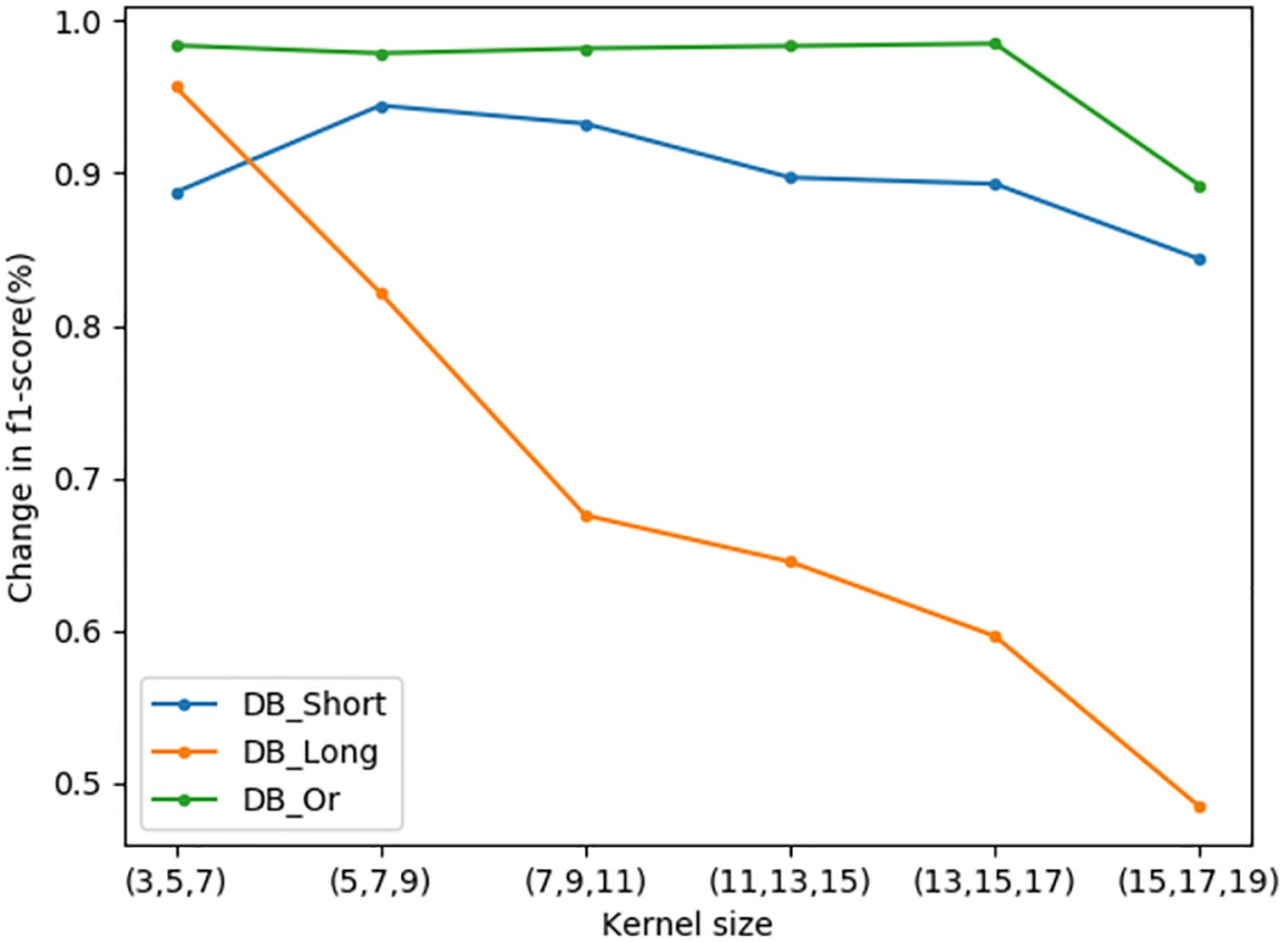
The effect of convolution kernel size.

**Fig 12:**
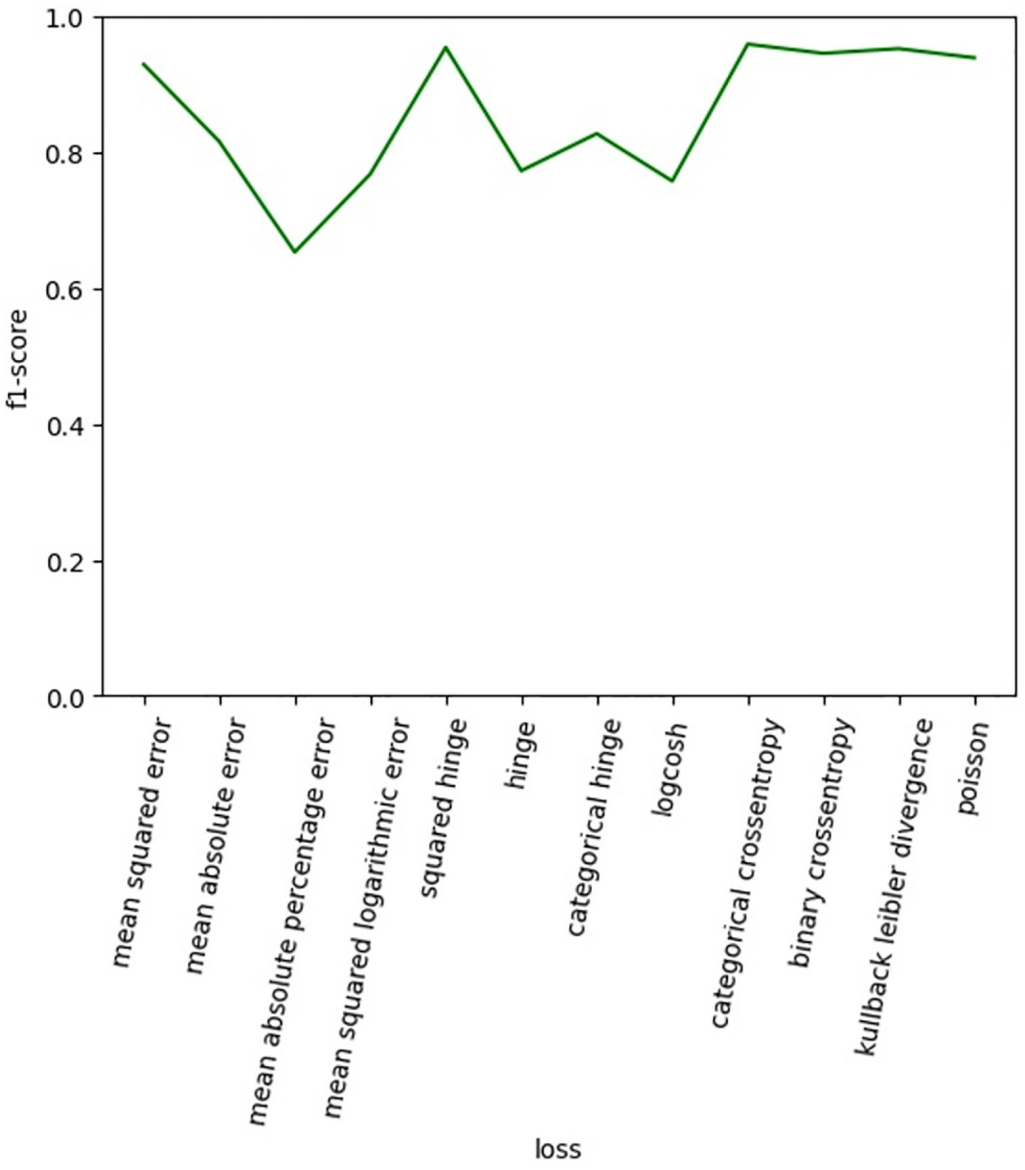
The impact of different loss.

**Fig 13:**
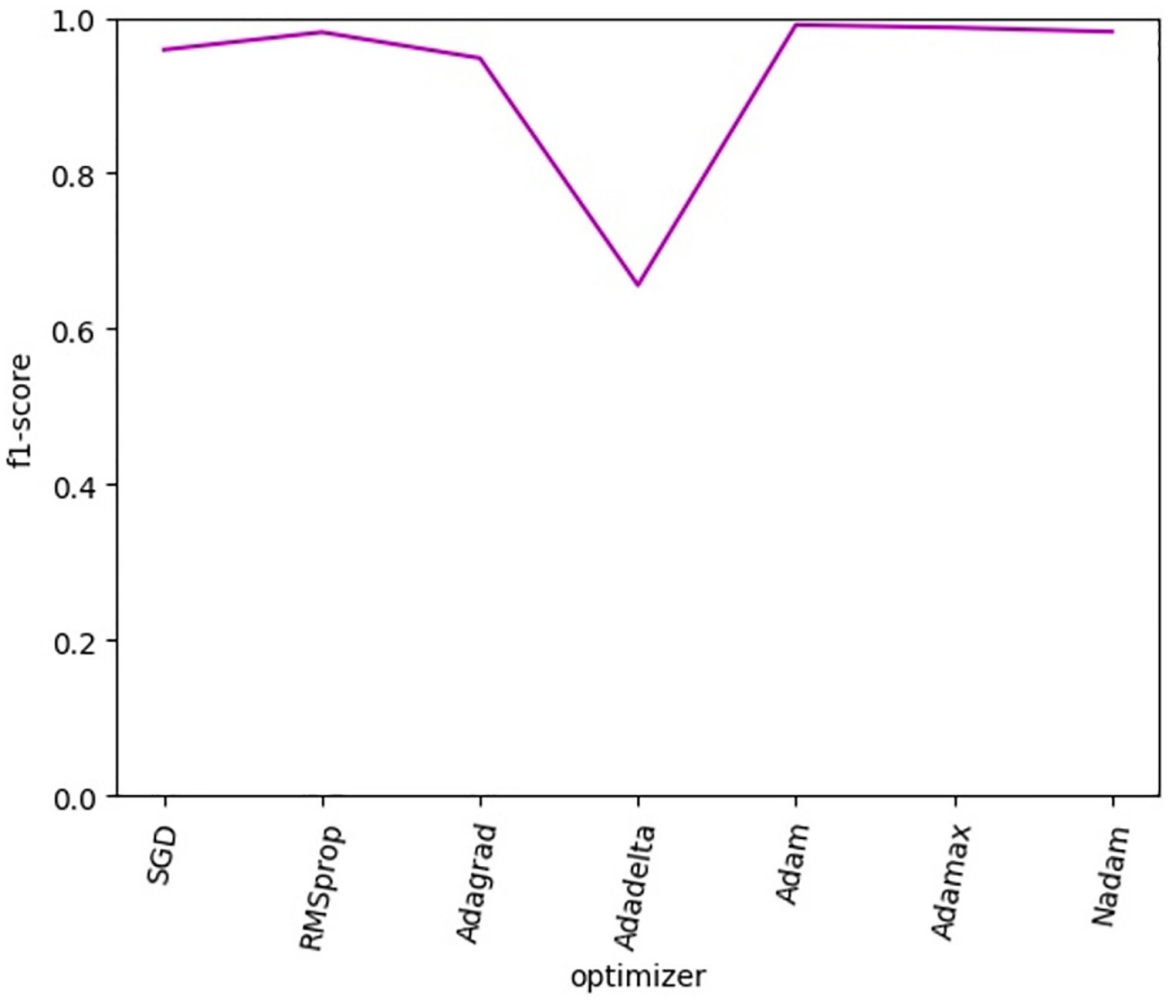
Effects of different optimizers.

After the filter size was set to 32, six groups of combined convolution kernels were used for experiments, and the experimental results were shown in Fig 11. In DB_Or dataset, it can be found that the convolution kernel size has little influence on it. In DB_Long dataset, the best effect is achieved in the group of convolution kernels (3,5,7), and then decreases with the increase of the convolution kernels. In DB_Shor dataset, the best effect is achieved in the group of convolution kernels (5,7,9), and then decreases with the increase of the convolution kernels. After comprehensive consideration, we finally use the convolution kernel (3,5,7). In order to select a better loss function, this paper uses the mainstream loss function to carry out a comparative experiment. The experimental results are shown in Fig 12. By comparison, we can see that the result is best when loss is categorical crossentropy, so the loss function in this paper is chosen as categorical crossentropy. In order to select a better optimizer, this paper uses the mainstream optimizer to carry out a comparative experiment. The experimental results are shown in Fig 13. Through comparison, it is found that when the optimizer is Nadam, the effect is the best, so the optimizer in this paper is Nadam.

In order to verify that residual connection in RDCNet can solve the problem of short-distance feature loss and gradient disappearance, RDCNet connecting BiLSTM can solve the problem of long-distance association feature capture, BiLSTM connecting Transfrom can solve the problem of BiLSTM with the same authority information, this paper conducted ablation experiments. The experimental results are shown in Table 3. Among them, JACLNet denotes the method of this paper, JACLnet-No-Rest denotes the experimental results that RDCNet in JACLNet does not use residual connection, JACLnet-No-BiLSTM denotes the experimental results that JACLNet does not use BiLSTM, JACLnet-No-Transfrom represents the experimental result that JACLNet does not use Transfrom. Through experimental comparison, it can be found that the *F*1-*score* value of JACLNet-No-Rest on DB_Short dataset has the largest difference with JACLNet, because the data in DB_Shor dataset is short code, and in the process of DB_Train training, With the deepening of depth, the shorter sequence association feature information will be weakened in the long text. The *Recall* value of JACLNet-NO-Rest is very low, because the malicious code in the dataset is mostly short code (only a few hundred KB at most). Due to the short sequence, the short sequence information will be weakened with the deepening of depth, resulting in the reduction of *Recall* value. JACLNet-NO-BiLSTM in DB_Long dataset, the *F*1-*score* value and *Recall* value are both very low, because RDCNet cannot obtain the association information of too long sequence, and BiLSTM mainly captures the association between the short-distance relationship information obtained by RDCNet. If you do not add BiLSTM after RDCNet, long JavaScript code will be poorly detected. The *F*1-*score* of JACLNet-NO-Transfrom on these three datasets is reduced to varying degrees, because Transfrom mainly recalculates the feature weight of the text. Especially on DB_Long dataset, the *F*1-*score* value of JACLNet-NO-Transfrom is very low, because BiLSTM uses the same weight for training when acquiring long-distance features, which leads to that the important correlation features between short-distance features obtained in RDCNet are not particularly obvious. The final detection effect is relatively poor. The MCC value on DB_Long and DB_Short data is low, which is because the amount of malicious code is different from that of benign code during training, resulting in data imbalance.

**Table 3:** Ablation experiment.

Dataset	Model	*Accuracy*	*Precision*	*Recall*	*FPR*	*F*1-*score*	*MCC*
DB_Long	JACLNet-NO-Rest	94.35%	94.00%	95.40%	6.81%	94.69%	84.07%
	JACLNet-NO-BiLSTM	97.81%	99.99%	96.14%	0.01%	98.03%	90.79%
	JACLNet-NO-Transfrom	89.53%	99.70%	83.83%	0.44%	91.07%	57.48%
	**JACLNet**	**97.63%**	**99.41%**	**96.42%**	**0.75%**	**97.89%**	**90.78%**
DB_Short	JACLNet-NO-Rest	90.38%	99.26%	83.70%	0.81%	90.82%	64.44%
	JACLNet-NO-BiLSTM	91.73%	99.70%	83.83%	0.26%	91.07%	70.71%
	JACLNet-NO-Transfrom	95.05%	99.31%	90.04%	0.54%	94.44%	82.49%
	**JACLNet**	**95.11%**	**99.45%**	**90.04%**	**0.43%**	**94.51%**	**82.57%**
DB_Or	JACLNet-NO-Rest	98.23%	98.74%	97.13%	0.93%	97.92%	94.31%
	JACLNet-NO-BiLSTM	98.33%	99.27%	97.19%	0.64%	97.19%	94.22%
	JACLNet-NO-Transfrom	97.97%	98.86%	96.83%	1.00%	97.83%	93.22%
	**JACLNet**	**98.90%**	**99.42%**	**98.25%**	**0.51%**	**98.83%**	**96.27%**

In this paper, the current mainstream deep learning model(Among them, deep learning methods BiLSTM+Attention [21], DPCNN+BiLSTM [22] and JSContana [23] in this laboratory have some differences in experimental results with original results due to differences in data pretreatment, dataset and equipment.), traditional machine learning method and mainstream antivirus engine, compare with the proposed method on the DB_Or dataset by using the 5-fold cross-validation method. In addition, all detection models used *Accuracy*, *Precision*, *Recall*, *FPR*, *F*1-*score* and *MCC* to measure the detection results. The experimental comparison results are shown in Table 4. As can be seen from the table, the detection effect of JACLNet method in this paper is better than other methods. On the DB_Or dataset, JACLNet’s *Accuracy* reaches 98.90%, indicating that this method can accurately find most malicious codes in the test process. At the same time, the *Precision* of the model reaches 99.42%, indicating that the method can accurately find most benign codes in the testing process. This is because BiLSTM network and Transfrom are added to JACLNet in this paper. Since benign codes are mainly longer texts, BiLSTM can be added to obtain long-distance associated features, and Transfrom can be added to repeatedly weight the obtained long-distance features, so as to improve the ability of benign code detection. The *Recall* of JACLNet model is 98.25%, and the *FPR* is 0.51%, indicating that the JACLNET model causes very few false positives. This is because RDCNet network is added to JACLNet in this paper. Since malicious code is mainly short text, adding RDCNet can accurately obtain the characteristics of short text, and thus reduce the phenomenon of false positives. The *F*1-*score* of JACLNet reaches 98.83%, indicating that the model in this paper achieves a higher true positive rate and lower false positive rate. The *MCC* of JACLNet reaches 96.27%, which indicates that the detection effect of the proposed model is better than other models when the data are not balanced.

**Table 4:** The model compares the experimental results.

Dataset	Model	*Accuracy*	*Precision*	*Recall*	*FPR*	*F*1-*score*	*MCC*
DB_Or	BiLSTM+Attention	96.47%	97.28%	95.74%	2.76%	96.50%	88.86%
	DPCNN+BiLSTM	97.11%	99.46%	95.01%	0.56%	97.17%	89.14%
	JSContana	97.47%	98.77%	96.26%	1.25%	97.50%	91.23%
	**JACLNet**	**98.90%**	**99.42%**	**98.25%**	**0.51%**	**98.83%**	**96.27%**
	SVM	96.56%	98.77%	94.59%	1.27%	96.63%	87.70%
	Random Forest	97.47%	95.87%	99.05%	4.00%	97.43%	94.15%
	Avast	95.82%	97.90%	94.00%	2.19%	95.91%	85.73%
	ClamAV	92.54%	97.89%	88.43%	2.36%	92.91%	73.23%
	Avira	95.97%	97.85%	94.31%	2.23%	96.05%	86.35%

In order to verify that the abstract syntax tree recombination algorithm proposed in this paper can achieve the effect of data enhancement and provide rich syntax information for subsequent model training, a comparative experiment before and after abstract syntax tree recombination is conducted in this paper. The experimental results are shown in Table 5. Through the experimental results, it can be found that after the implementation of the abstract syntax tree recombination algorithm (DB_Re), the model detection results of each index are higher than the detection results of the model without the implementation of the abstract syntax tree recombination algorithm (DB_Or). In BiLSTM+Attention, the difference of *F*1-*score* before and after using the abstract syntax tree recombination algorithm reaches 1.72%. This is because the abstract syntax tree recombination algorithm proposed in this paper can achieve the effect of data enhancement, which can improve the generalization ability and robustness of the model in model training, so the detection results have been significantly improved after using the abstract syntax tree recombination algorithm.

**Table 5:** Experimental results of model comparison before and after abstract syntax tree recombination.

Dataset	Model	*Accuracy*	*Precision*	*Recall*	*FPR*	*F*1-*score*	*MCC*
DB_Or	BiLSTM+Attention	96.13%	98.69%	93.89%	1.38%	96.23%	86.12%
	DPCNN+BiLSTM	97.97%	99.11%	96.91%	0.91%	97.99%	92.86%
	JSContana	97.41%	98.39%	96.49%	1.64%	97.43%	91.38%
	**JACLNet**	**98.90%**	**99.42%**	**98.25%**	**0.51%**	**98.83%**	**96.27%**
DB_Re	BiLSTM+Attention	97.97%	99.82%	96.15%	0.16%	97.95%	92.26%
	DPCNN+BiLSTM	98.25%	98.04%	98.52%	2.01%	98.28%	95.05%
	JSContana	98.93%	98.97%	98.91%	1.04%	98.93%	96.77%
	**JACLNet**	**99.60%**	**99.37%**	**99.83%**	**0.62%**	**99.83%**	**99.94%**

In order to verify that the method in this paper can effectively detect JavaScript malicious codes that are too long or too short, DB_Train is used for training in this paper, and tests are carried out on DB_Long and DB_Short datasets. The experimental comparison results are shown in Table 6. On the DB_Long dataset, the *Recall* of the proposed JACLNet method reaches 96.42%, which is 2.88% higher than that of JSContana, indicating that the proposed method is superior to the current mainstream deep learning model in long-distance JavaScript code detection. On the DB_Short dataset, the *Recall* of the proposed method is only 90.04%, but the proposed method is the best among these deep learning methods. Where ASR+JACLNet represents the model trained after using the abstract syntax tree reorganization algorithm, ASR+JACLNet *F*1-*score*reaches 98.87% on the DB_Long dataset, On DB_Short dataset, *F*1-*score*reaches 97.32%, which is improved by 0.98% and 2.81%, respectively, compared with that without using abstract syntax tree recombination algorithm.

**Table 6:** Comparison of long code and short code test results.

Dataset	Model	*Accuracy*	*Precision*	*Recall*	*FPR*	*F*1-*score*	*MCC*
DB_Long	BiLSTM+Attention	94.18%	96.73%	92.04%	0.03%	94.32%	80.65%
	DPCNN+BiLSTM	90.53%	99.11%	84.59%	1.06%	91.28%	64.27%
	JSContana	96.23%	99.34%	93.54%	0.70%	96.35%	85.87%
	**JACLNet**	**97.63%**	**99.41%**	**96.42%**	**0.75%**	**97.89%**	**90.78%**
	**ASR+JACLNet**	**98.72%**	**99.87%**	**97.88%**	**0.16%**	**98.87%**	**94.72%**
DB_Short	BiLSTM+Attention	85.99%	99.63%	78.28%	0.50%	87.67%	46.30%
	JSContana	88.46%	98.65%	81.96%	1.69%	89.53%	57.00%
	DPCNN+BiLSTM	83.43%	0.99%	75.12%	0.014%	85.79%	35.78%
	**JACLNet**	**95.11%**	**99.45%**	**90.04%**	**0.43%**	**94.51%**	**82.57%**
	**ASR+JACLNet**	**97.03%**	**99.86%**	**94.91%**	**0.16%**	**97.32%**	**87.81%**

**Fig 14:**
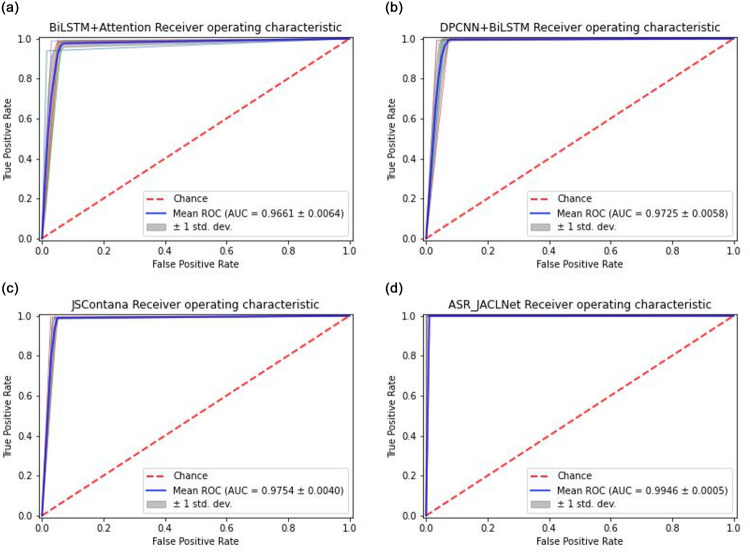
ROC curve comparison diagram. (a) BiLSTM+Attention. (b) DPCNN+BiLSTM. (c) JSContana. (d) ASR+JACLNet.

In order to further explore the effect of the method proposed in this paper, the comparison experiment was conducted again using the 5-fold cross-validation method under the same environment and parameters. ROC Curves and AUC scores of each model are shown in Fig 14. Where, ASR+JACLNet represents the JACLNet that is trained after using the abstract syntax tree reorganization algorithm proposed in this paper. From the ROC curve, it can be seen that the range between the ROC curves of the JACLNet proposed in this work is much smaller than that of other models and the AUC score is higher than that of other models, indicating that the method proposed in this work is stable in detecting malicious code and has high accuracy.

**Fig 15:**
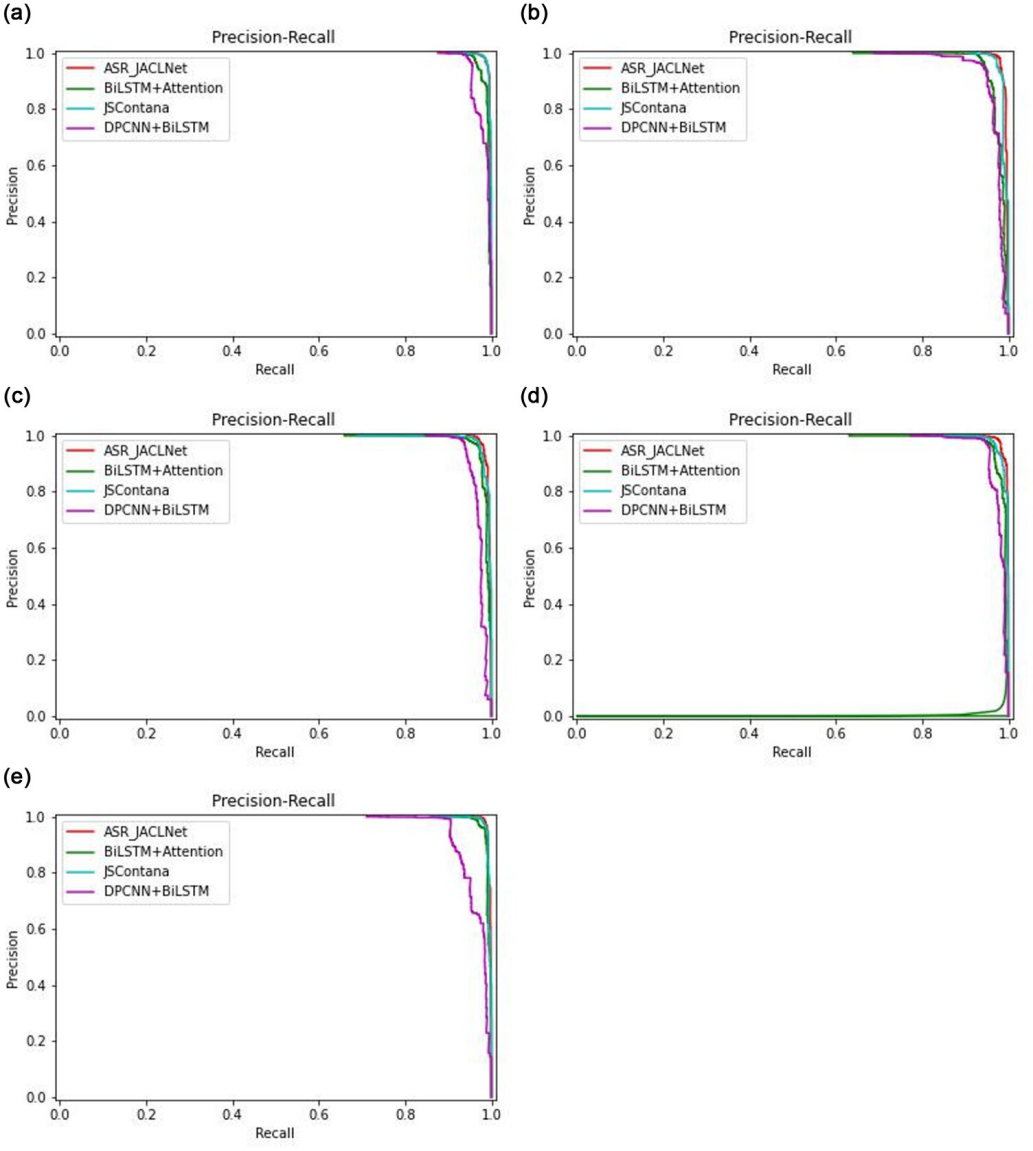
ROC curve comparison diagram. (a) The result of the first fold PR curve. (b) The result of the second fold PR curve. (c) The result of the third fold PR curve. (d) The result of the fourth fold PR curve. (e) The result of the fifth fold PR curve.

When the distribution of positive and negative samples in the test set changes, the ROC curve basically remains unchanged. In order to reflect the detection effect of JACLNet model when the distribution of positive and negative samples is different in the test set, the comparative experiment of PR curve is conducted in this paper. In this paper, we use 5-fold cross validation in the experiment, because cross validation will make the distribution of positive and negative samples in the test set somewhat different. This is shown in Fig 15. Through these five different tests, it can be found that the PR curve result of the proposed method ASR_JACLNet is the closest to the right each time, and it falls vertically, which indicates that the proposed method performs the best. In the first, third and fourth, comparisons, when *Recall* is 0, *Precision* is not close to 100% for BiLSTM+Attention, DPCNN+BiLSTM and JSContana, while the proposed method can be close to 100%. It shows that the detection effect of the method in this paper is still considerable when the distribution of positive and negative samples is different.

Because malicious code detection is mainly applied to real-time network detection, there are certain requirements for detection speed. In model training and detection, this paper records the processing time of the model on training samples and test samples, as shown in Table 7. The loading and prediction time of JACLNe model is longer than that of other deep learning models, but in the actual environment, the detection time can meet the requirements of real-time detection.

**Table 7:** The experimental results were compared with the model time.

Model	Training time	Loading time	The test of time
BiLSTM-Attention	15.46min	0.98s	4.80ms
DPCNN+BiLSTM	2.05min	0.99s	1.44ms
**JACLNet**	**2.51min**	**1.63s**	**2.01ms**
SVM	1.46min	0.01s	0.14ms
Random Forest	0.89min	0.04s	0.05ms

## Conclusion

In this paper, in order to solve the problem that the current deep learning methods are poor at detecting too long or too short JavaScript codes, this paper proposes a deep learning network with an adaptive code length, JACLNet, which is used to capture the association features of variable distance between codes. In order to provide rich syntax information for subsequent feature extraction, this paper proposes a syntax tree recombination algorithm to achieve code enhancement. Finally, this paper compares with the current mainstream detection model, and the detection effect is significantly improved.

Compared with the current mainstream methods, the proposed method has a high detection rate. Although the proposed method can achieve the real-time detection effect, the detection effect is longer than that of the traditional machine learning method, which needs to be optimized in the later stage. In addition, RDCNet is designed in this model to solve the short-distance feature capture problem, and BiLSTM and Transfrom are used in this model to solve the long-distance feature problem, which leads to the complex structure of the model. How to design a concise model for variable distance detection is the focus of future research.
